# Adaptation of the Human Eye to Distances

**Published:** 1860-01

**Authors:** Charles Archer

**Affiliations:** Surgeon in the Bengal Army


### Selections.

### ADAPTATION OF THE HUMAN EYE TO DISTANCES.

BY CHARLES ARCHER,

Surgeon in the Bengal Army.

The following is a summary of the author's views :—

1. The eye is adapted to varying distances principally by an alteration in the fibrous arrangement of the lens itself. Moreover, that when the lens is removed after an operation this power is nearly lost, and can only be exerted within very confined distances.

2. The purpose of focalizing light at short distances is doubtless assisted, as suggested by Bowman, by the contractions of the ciliary muscle, in its antero-posterior direction, bringing forward the ciliary process.

3. As the posterior hemisphere of the capsule is firmly united to the hyaloid membrane, this portion must always remain quiescent, and therefore the antero-posterior contractions of the ciliary muscle must be very limited as regards the lens.

4. The ciliary muscle being placed around the eye, and its fibres being of a somewhat plexiform character, the contractions of the muscle will relax those yielding portions of the eye placed within its circumference.

5. The relaxations of the ciliary processes will deprive the capsule of its firm support. It will be pressed forward by the lens, which will meet with no further resistance to the expansion of its short axis.

6. The lens itself, as microscopically described by Bowman and Kolliker, is admirably adapted to the varying changes which take place in the capsule.

7. The posterior capsule being firmly united to the hyaloid membrane, the alteration in the diameters of the cavity of the capsule must take place from the periphery of the lens to its centre, and from behind forward, but not from before backwards, on account of the close union of the posterior capsule to the hyaloid membrane.

8. To allow such alterations to take place without endangering the achromatism of the lens, the alterations in the plane of its long diameter must be synchronous with the alterations in the plane of its short diameter. To allow of this, the margin of the lens is free in the canal of Petit; were this not the case, chromatic aberration would result.

9. The elasticity of the capsule of the lens and the ciliary muscle are antagonistic; that on the ciliary muscle becoming relaxed, the capsule of the lens is free to exert that elasticity.

10. By the pressure exerted by the anterior hemisphere of the capsule, by means of the polygonal cells of Virchow, on the anterior face of the lens, the organ is able to fulfil all the requirements for adapting it to receive focalized light from long distances.

11. The polygonal cells of Virchow are placed on the posterior surface of the anterior hemisphere of the capsule with the view before mentioned, and they are arranged with their long diameters in an antero-posterior direction, that pressure may not injure their transparency, which would be the case if placed laterally.

12. These cells are not found in other parts of the capsule.

13. The fibres of the lens are serrated for the purpose of uniting either to the other, so as to allow them freedom of motion without altering their ultimate relations to each other.

14. The ciliary muscle is very highly endowed with nervous matter to supply all these varying requirements.

15. By the above postules, all the modern discoveries in the microscopical anatomy of the eye receive a distinct expression of their individual functions, and by so doing, adapt the organ of vision to the acknowledged laws of light.

